# *N*-(4-Meth­oxy­phen­yl)benzene­sulfonamide

**DOI:** 10.1107/S1600536811000365

**Published:** 2011-01-08

**Authors:** Saba Ibrahim, M. Nawaz Tahir, Nadeem Iqbal, Durre Shahwar, Muhammad Asam Raza

**Affiliations:** a Department of Chemistry, Government College University, Lahore, Pakistan; b Department of Physics, University of Sargodha, Sargodha, Pakistan

## Abstract

In the title compound, C_13_H_13_NO_3_S, the benzene ring of the benzene­sulfonamide moiety is disordered with an occupancy ratio of 0.56 (3):0.44 (3), the disorder components being twisted at and angle of 21 (1)° to each other. The meth­oxy­benzene group is roughly planar (r.m.s. deviation = 0.0144 Å) and the amide N atom is displaced from this plane by 0.090 (6) Å. The dihedral angles between the meth­oxy­benzene group and the major and minor occupancy components of the disordered benzene ring are 54.6 (4) and 62.9 (5)°, respectively. In the crystal, infinite polymeric chains are formed along [100] due to inter­molecular N—H⋯O hydrogen bonding. Weak C—H⋯π inter­actions  are also present in the crystal.

## Related literature

For related structures, see: Kato *et al.* (2006 ▶); Perlovich *et al.* (2009 ▶).
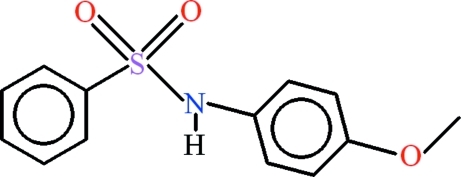


## Experimental

### 

#### Crystal data

C_13_H_13_NO_3_S
*M* *_r_* = 263.30
Orthorhombic, 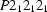

*a* = 5.3094 (5) Å
*b* = 8.5309 (10) Å
*c* = 27.925 (3) Å
*V* = 1264.8 (2) Å^3^
*Z* = 4
Mo *K*α radiation
μ = 0.26 mm^−1^
*T* = 296 K
0.30 × 0.14 × 0.12 mm

#### Data collection

Bruker Kappa APEXII CCD diffractometer
Absorption correction: multi-scan (*SADABS*; Bruker, 2005 ▶) *T*_min_ = 0.961, *T*_max_ = 0.970
7272 measured reflections
2457 independent reflections
1503 reflections with *I* > 2σ(*I*)
*R*_int_ = 0.060

#### Refinement

*R*[*F* ^2^ > 2σ(*F* ^2^)] = 0.061
*wR*(*F* ^2^) = 0.131
*S* = 1.02
2457 reflections
138 parameters
1 restraint
H atoms treated by a mixture of independent and constrained refinement
Δρ_max_ = 0.41 e Å^−3^
Δρ_min_ = −0.28 e Å^−3^
Absolute structure: Flack (1983 ▶), 961 Friedel pairs
Flack parameter: 0.09 (16)

### 

Data collection: *APEX2* (Bruker, 2009 ▶); cell refinement: *SAINT* (Bruker, 2009 ▶); data reduction: *SAINT*; program(s) used to solve structure: *SHELXS97* (Sheldrick, 2008 ▶); program(s) used to refine structure: *SHELXL97* (Sheldrick, 2008 ▶); molecular graphics: *ORTEP-3 for Windows* (Farrugia, 1997 ▶) and *PLATON* (Spek, 2009 ▶); software used to prepare material for publication: *WinGX* (Farrugia, 1999 ▶) and *PLATON*.

## Supplementary Material

Crystal structure: contains datablocks global, I. DOI: 10.1107/S1600536811000365/bq2272sup1.cif

Structure factors: contains datablocks I. DOI: 10.1107/S1600536811000365/bq2272Isup2.hkl

Additional supplementary materials:  crystallographic information; 3D view; checkCIF report

## Figures and Tables

**Table 1:** Hydrogen-bond geometry (Å, °) *Cg*1, *Cg*2 and *Cg*3 are the centroids of the C1*A*–C6*A*, C7–C12 and C1*B*–C6*B* rings, respectively.

*D*—H⋯*A*	*D*—H	H⋯*A*	*D*⋯*A*	*D*—H⋯*A*
N1—H1⋯O2^i^	0.83 (2)	2.22 (2)	3.039 (4)	170 (4)
C8—H8⋯*Cg*2^ii^	0.93	2.93	3.613 (5)	132
C13—H13*B*⋯*Cg*1^iii^	0.96	2.98	3.766 (6)	140
C13—H13*B*⋯*Cg*3^iii^	0.96	2.96	3.763 (7)	143

Symmetry codes: (i) 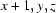; (ii) 
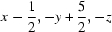
; (iii) 
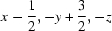
.

**Fig. 1.**
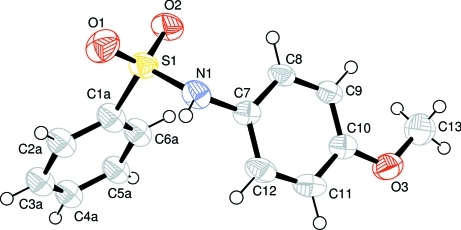
View of the title compound with the atom numbering scheme having atoms of greater occupancy ratio. The thermal ellipsoids are drawn at the 50% probability level.

**Fig. 2.**
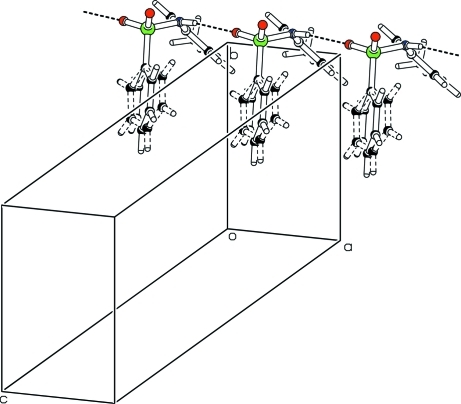
The partial packing (PLATON; Spek, 2009) which shows that molecules form polymeric chains extending along the a axis.

## supplementary crystallographic information

### Comment

The title compound (I, Fig. 1) is a part of the synthesis of sulfonamides and consequently the study of their bioactivity. The crystal structures of 4-amino-N-(4-methoxyphenyl)benzenesulfonamide (Perlovich *et al.*, 2009) and P-(+)-N-Phenyl-4-methoxybenzenesulfonamide (Kato, *et al.*, 2006) have been published previously which are related to the title compound (I).

In (I), the phenyl ring of benzenethiol moiety is disordered over two set of sites A (C1A—C6A) and B (C1B—C6B) with occupancy ratio of 0.56 (3):0.44 (3). The dihedral angle between A/B is 21 (1)°. The methoxybenzene group C (C7—C13/O2) is almost planar with r. m. s. deviation of 0.0144 Å and amide atom N1 is at a distance of  0.0897 (55)Å. The dihedral angle between A/C and B/C is 54.63 (35)° and 62.86 (50)°, respectively. The sulfonyl group D (S1/O1/O2) is of course planar. The dihedral angles between A/D, B/D and C/D are 53.37 (43)°, 51.65 (50)° and 24.10 (28)°, respectively. The molecules are stabilized in the form of infinite one-dimensional polymeric chains due to N—H···O type (Table 1, Fig. 2) extending along the crystallographic *a*-axis. The C—H···π interactions (Table 1) also play important role in stabilizing the molecules.

### Experimental

Equal molar (10 mmol) quantity of benzene sulfonyl chloride and *para* anisidine was mixed in 10 ml distilled water under stirring at room temperature. During the reaction pH was adjusted at 8 using dilute solution of sodium carbonate. The reaction was monitored using TLC. On the completion of reaction the pH was made acidified using 3 N HCl. The crude product was separated by filtration, dried and recrystalized in methanol to afford white needles of (I) after 72 h.

### Refinement

The benzene ring of benzenethiol moiety is disordered over two set of sites with occupancy ratio of 0.56 (3):0.44 (3). The rings are fitted in regular hexagons with nearly equal bond distances and bond angles. The thermal parameters of C-atoms within disordered benzene rings are treated to be equal.

The coordinates of amide H-atom were refined. All other H-atoms were positioned geometrically (C–H = 0.93–0.96 Å) and refined as riding with *U*_iso_(H) = *xU*_eq_(C, N), where *x* = 1.5 for methyl H-atoms and *x* = 1.2 for all other H-atoms.

### Crystal data

**Table:**

C_13_H_13_NO_3_S	*F*(000) = 552
*M**_r_* = 263.30	*D*_x_ = 1.383 Mg m^−^^3^
Orthorhombic, *P*2_1_2_1_2_1_	Mo *K*α radiation, λ = 0.71073 Å
Hall symbol: P 2ac 2ab	Cell parameters from 1503 reflections
*a* = 5.3094 (5) Å	θ = 2.5–25.2°
*b* = 8.5309 (10) Å	µ = 0.26 mm^−^^1^
*c* = 27.925 (3) Å	*T* = 296 K
*V* = 1264.8 (2) Å^3^	Needle, white
*Z* = 4	0.30 × 0.14 × 0.12 mm

### Data collection

**Table:**

Bruker Kappa APEXII CCD diffractometer	2457 independent reflections
Radiation source: fine-focus sealed tube	1503 reflections with *I* > 2σ(*I*)
graphite	*R*_int_ = 0.060
Detector resolution: 8.00 pixels mm^-1^	θ_max_ = 26.0°, θ_min_ = 2.5°
ω scans	*h* = −4→6
Absorption correction: multi-scan (*SADABS*; Bruker, 2005)	*k* = −9→10
*T*_min_ = 0.961, *T*_max_ = 0.970	*l* = −32→31
7272 measured reflections	

### Refinement

**Table:**

Refinement on *F*^2^	Secondary atom site location: difference Fourier map
Least-squares matrix: full	Hydrogen site location: inferred from neighbouring sites
*R*[*F*^2^ > 2σ(*F*^2^)] = 0.061	H atoms treated by a mixture of independent and constrained refinement
*wR*(*F*^2^) = 0.131	*w* = 1/[σ^2^(*F*_o_^2^) + (0.0506*P*)^2^ + 0.2719*P*] where *P* = (*F*_o_^2^ + 2*F*_c_^2^)/3
*S* = 1.02	(Δ/σ)_max_ < 0.001
2457 reflections	Δρ_max_ = 0.41 e Å^−^^3^
138 parameters	Δρ_min_ = −0.28 e Å^−^^3^
1 restraint	Absolute structure: Flack (1983), 961 Friedel pairs
Primary atom site location: structure-invariant direct methods	Flack parameter: 0.09 (16)

### Special details

**Table:**

Geometry. Bond distances, angles *etc*. have been calculated using the rounded fractional coordinates. All su's are estimated from the variances of the (full) variance-covariance matrix. The cell e.s.d.'s are taken into account in the estimation of distances, angles and torsion angles
Refinement. Refinement of *F*^2^ against ALL reflections. The weighted *R*-factor *wR* and goodness of fit *S* are based on *F*^2^, conventional *R*-factors *R* are based on *F*, with *F* set to zero for negative *F*^2^. The threshold expression of *F*^2^ > σ(*F*^2^) is used only for calculating *R*-factors(gt) *etc*. and is not relevant to the choice of reflections for refinement. *R*-factors based on *F*^2^ are statistically about twice as large as those based on *F*, and *R*- factors based on ALL data will be even larger.

### Fractional atomic coordinates and isotropic or equivalent isotropic displacement parameters (Å^2^)

**Table:**

	*x*	*y*	*z*	*U*_iso_*/*U*_eq_	Occ. (<1)
S1	0.5888 (2)	1.17782 (15)	0.14716 (4)	0.0434 (4)	
O1	0.6723 (6)	1.2938 (4)	0.18015 (10)	0.0615 (14)	
O2	0.3371 (4)	1.1850 (4)	0.12785 (9)	0.0549 (13)	
O3	0.7036 (6)	0.8660 (4)	−0.07098 (11)	0.0570 (12)	
N1	0.7813 (6)	1.1858 (5)	0.10190 (12)	0.0427 (14)	
C1A	0.6261 (14)	0.9927 (7)	0.1750 (2)	0.0500 (19)	0.56 (3)
C2A	0.7618 (19)	0.9727 (8)	0.2171 (3)	0.0500 (19)	0.56 (3)
C3A	0.786 (2)	0.8243 (10)	0.2371 (2)	0.0500 (19)	0.56 (3)
C4A	0.6743 (17)	0.6959 (8)	0.2150 (2)	0.0500 (19)	0.56 (3)
C5A	0.5386 (12)	0.7159 (8)	0.1729 (3)	0.0500 (19)	0.56 (3)
C6A	0.5145 (17)	0.8643 (9)	0.1529 (3)	0.0500 (19)	0.56 (3)
C7	0.7490 (7)	1.0996 (5)	0.05846 (15)	0.0353 (16)	
C8	0.5531 (8)	1.1329 (5)	0.02810 (14)	0.0403 (16)	
C9	0.5314 (8)	1.0566 (5)	−0.01519 (16)	0.0413 (17)	
C10	0.7056 (8)	0.9465 (6)	−0.02856 (16)	0.0407 (17)	
C11	0.9050 (9)	0.9139 (5)	0.00183 (17)	0.0490 (17)	
C12	0.9241 (9)	0.9870 (6)	0.04544 (16)	0.0473 (16)	
C13	0.4974 (11)	0.8934 (7)	−0.10251 (17)	0.075 (3)	
C3B	0.879 (3)	0.8430 (11)	0.2267 (4)	0.054 (3)	0.44 (3)
C4B	0.726 (2)	0.7133 (10)	0.2186 (3)	0.054 (3)	0.44 (3)
C5B	0.5220 (14)	0.7245 (11)	0.1876 (6)	0.054 (3)	0.44 (3)
C6B	0.4708 (16)	0.8655 (12)	0.1646 (5)	0.054 (3)	0.44 (3)
C2B	0.827 (3)	0.9840 (9)	0.2037 (5)	0.054 (3)	0.44 (3)
C1B	0.6235 (18)	0.9952 (10)	0.1726 (3)	0.054 (3)	0.44 (3)
H6A	0.42369	0.87769	0.12470	0.0601*	0.56 (3)
H5A	0.46390	0.62998	0.15812	0.0601*	0.56 (3)
H11	1.02714	0.84188	−0.00742	0.0589*	
H12	1.05444	0.96101	0.06623	0.0569*	
H13A	0.34217	0.88310	−0.08517	0.1122*	
H13B	0.50147	0.81823	−0.12809	0.1122*	
H13C	0.50967	0.99726	−0.11552	0.1122*	
H8	0.43398	1.20759	0.03680	0.0480*	
H9	0.39767	1.08001	−0.03547	0.0494*	
H1	0.930 (4)	1.197 (5)	0.1105 (13)	0.0514*	
H2A	0.83652	1.05863	0.23189	0.0601*	0.56 (3)
H3A	0.87672	0.81092	0.26532	0.0601*	0.56 (3)
H4A	0.69041	0.59660	0.22843	0.0601*	0.56 (3)
H2B	0.92955	1.07075	0.20904	0.0646*	0.44 (3)
H3B	1.01505	0.83549	0.24742	0.0646*	0.44 (3)
H4B	0.76021	0.61899	0.23400	0.0646*	0.44 (3)
H5B	0.41988	0.63775	0.18221	0.0646*	0.44 (3)
H6B	0.33438	0.87301	0.14384	0.0646*	0.44 (3)

### Atomic displacement parameters (Å^2^)

**Table:**

	*U*^11^	*U*^22^	*U*^33^	*U*^12^	*U*^13^	*U*^23^
S1	0.0335 (6)	0.0474 (8)	0.0494 (7)	0.0026 (6)	−0.0036 (5)	−0.0007 (7)
O1	0.060 (2)	0.060 (3)	0.0646 (19)	−0.0021 (18)	−0.0064 (16)	−0.022 (2)
O2	0.0254 (16)	0.075 (3)	0.0642 (18)	0.0085 (17)	−0.0046 (13)	0.004 (2)
O3	0.056 (2)	0.057 (2)	0.058 (2)	0.0110 (17)	0.0010 (17)	−0.0093 (18)
N1	0.0250 (19)	0.054 (3)	0.049 (2)	−0.005 (2)	−0.0056 (17)	0.005 (2)
C1A	0.042 (3)	0.056 (4)	0.052 (3)	−0.004 (2)	−0.001 (2)	0.014 (2)
C2A	0.042 (3)	0.056 (4)	0.052 (3)	−0.004 (2)	−0.001 (2)	0.014 (2)
C3A	0.042 (3)	0.056 (4)	0.052 (3)	−0.004 (2)	−0.001 (2)	0.014 (2)
C4A	0.042 (3)	0.056 (4)	0.052 (3)	−0.004 (2)	−0.001 (2)	0.014 (2)
C5A	0.042 (3)	0.056 (4)	0.052 (3)	−0.004 (2)	−0.001 (2)	0.014 (2)
C6A	0.042 (3)	0.056 (4)	0.052 (3)	−0.004 (2)	−0.001 (2)	0.014 (2)
C7	0.025 (2)	0.034 (3)	0.047 (3)	−0.0053 (19)	0.005 (2)	0.004 (2)
C8	0.026 (2)	0.046 (3)	0.049 (3)	0.007 (2)	−0.003 (2)	0.007 (2)
C9	0.036 (3)	0.041 (3)	0.047 (3)	0.006 (2)	−0.003 (2)	0.007 (2)
C10	0.033 (3)	0.037 (3)	0.052 (3)	−0.004 (2)	0.002 (2)	0.003 (2)
C11	0.035 (3)	0.041 (3)	0.071 (3)	0.006 (2)	0.000 (3)	0.005 (3)
C12	0.028 (2)	0.053 (3)	0.061 (3)	0.003 (2)	−0.006 (2)	0.010 (3)
C13	0.088 (5)	0.081 (5)	0.055 (3)	0.018 (3)	−0.017 (3)	−0.017 (3)
C3B	0.055 (4)	0.055 (5)	0.052 (4)	−0.021 (3)	−0.010 (3)	0.010 (3)
C4B	0.055 (4)	0.055 (5)	0.052 (4)	−0.021 (3)	−0.010 (3)	0.010 (3)
C5B	0.055 (4)	0.055 (5)	0.052 (4)	−0.021 (3)	−0.010 (3)	0.010 (3)
C6B	0.055 (4)	0.055 (5)	0.052 (4)	−0.021 (3)	−0.010 (3)	0.010 (3)
C2B	0.055 (4)	0.055 (5)	0.052 (4)	−0.021 (3)	−0.010 (3)	0.010 (3)
C1B	0.055 (4)	0.055 (5)	0.052 (4)	−0.021 (3)	−0.010 (3)	0.010 (3)

### Geometric parameters (Å, °)

**Table:**

S1—O1	1.423 (3)	C7—C12	1.385 (6)
S1—O2	1.442 (2)	C8—C9	1.378 (6)
S1—N1	1.627 (3)	C9—C10	1.370 (6)
S1—C1A	1.771 (6)	C10—C11	1.385 (6)
S1—C1B	1.722 (9)	C11—C12	1.372 (7)
O3—C10	1.369 (6)	C2A—H2A	0.9300
O3—C13	1.424 (6)	C2B—H2B	0.9300
N1—C7	1.429 (6)	C3A—H3A	0.9300
N1—H1	0.83 (2)	C3B—H3B	0.9300
C1A—C6A	1.390 (10)	C4A—H4A	0.9300
C1A—C2A	1.389 (11)	C4B—H4B	0.9300
C1B—C2B	1.390 (18)	C5A—H5A	0.9300
C1B—C6B	1.390 (13)	C5B—H5B	0.9300
C2A—C3A	1.390 (11)	C6A—H6A	0.9300
C2B—C3B	1.391 (14)	C6B—H6B	0.9300
C3A—C4A	1.390 (11)	C8—H8	0.9300
C3B—C4B	1.391 (15)	C9—H9	0.9300
C4A—C5A	1.389 (10)	C11—H11	0.9300
C4B—C5B	1.390 (15)	C12—H12	0.9300
C5A—C6A	1.390 (11)	C13—H13B	0.9600
C5B—C6B	1.390 (16)	C13—H13C	0.9600
C7—C8	1.372 (6)	C13—H13A	0.9600
			
S1···H8	3.2000	C9···H8^viii^	3.0000
O1···C3A^i^	3.366 (9)	C10···H11^vi^	2.8200
O2···C8	3.045 (5)	C11···H11^vi^	2.9700
O2···N1^ii^	3.039 (4)	C13···H5A^v^	2.9300
O1···H4A^iii^	2.9200	C13···H9	2.5100
O1···H2B	2.4800	H1···O2^vii^	2.22 (2)
O1···H3A^iv^	2.8400	H1···H12	2.4500
O1···H3B^iv^	2.6400	H2A···O1	2.6200
O1···H2A	2.6200	H2B···O1	2.4800
O2···H1^ii^	2.22 (2)	H2B···H4B^iv^	2.3300
O2···H6A	2.6600	H2B···C4B^iv^	2.9800
O2···H8	2.6000	H2B···H3B^iv^	2.5800
O2···H6B	2.7000	H3A···O1^xi^	2.8400
O3···H5A^v^	2.8000	H3B···O1^xi^	2.6400
O3···H6A^v^	2.8200	H3B···H2B^xi^	2.5800
O3···H12^vi^	2.9000	H4A···O1^xii^	2.9200
N1···O2^vii^	3.039 (4)	H4A···C2A^ix^	3.0300
N1···H9^viii^	2.8000	H4B···C2B^xi^	3.0300
C2A···C4A^i^	3.547 (12)	H4B···H2B^xi^	2.3300
C3A···O1^ix^	3.366 (9)	H5A···O3^vi^	2.8000
C3B···C6B^vii^	3.594 (18)	H5A···C13^vi^	2.9300
C4A···C2A^ix^	3.547 (12)	H6A···O3^vi^	2.8200
C5A···C13^v^	3.266 (9)	H6A···O2	2.6600
C6A···C7	3.540 (9)	H6B···H13B^vi^	2.4500
C6B···C3B^ii^	3.594 (18)	H6B···O2	2.7000
C7···C9^viii^	3.509 (6)	H8···S1	3.2000
C7···C6A	3.540 (9)	H8···O2	2.6000
C8···C12^ii^	3.597 (6)	H8···C8^x^	3.0400
C8···O2	3.045 (5)	H8···C9^x^	3.0000
C9···C7^x^	3.509 (6)	H9···C13	2.5100
C9···C11^ii^	3.573 (6)	H9···H13A	2.2000
C10···C11^vi^	3.544 (7)	H9···H13C	2.4200
C11···C10^v^	3.544 (7)	H9···N1^x^	2.8000
C11···C9^vii^	3.573 (6)	H9···C7^x^	2.9200
C12···C8^vii^	3.597 (6)	H9···C8^x^	3.0600
C13···C5A^vi^	3.266 (9)	H11···C11^v^	2.9700
C2A···H4A^i^	3.0300	H11···C10^v^	2.8200
C2B···H4B^iv^	3.0300	H12···O3^v^	2.9000
C4A···H13B^v^	2.9900	H12···H1	2.4500
C4B···H13B^v^	2.9300	H13A···C9	2.6500
C4B···H2B^xi^	2.9800	H13A···C5A^vi^	3.0500
C5A···H13A^v^	3.0500	H13A···H9	2.2000
C5A···H13B^v^	2.7700	H13B···C4A^vi^	2.9900
C5B···H13B^v^	3.0600	H13B···C6A^vi^	3.1000
C6A···H13B^v^	3.1000	H13B···C4B^vi^	2.9300
C7···H9^viii^	2.9200	H13B···C5B^vi^	3.0600
C8···H8^viii^	3.0400	H13B···H6B^v^	2.4500
C8···H9^viii^	3.0600	H13B···C5A^vi^	2.7700
C9···H13C	2.8500	H13C···C9	2.8500
C9···H13A	2.6500	H13C···H9	2.4200
			
O1—S1—O2	120.1 (2)	C10—C11—C12	120.6 (4)
O1—S1—N1	106.2 (2)	C7—C12—C11	119.9 (4)
O1—S1—C1A	107.5 (2)	C1A—C2A—H2A	120.00
O1—S1—C1B	109.2 (3)	C3A—C2A—H2A	120.00
O2—S1—N1	106.85 (17)	C1B—C2B—H2B	120.00
O2—S1—C1A	107.8 (3)	C3B—C2B—H2B	120.00
O2—S1—C1B	107.0 (3)	C4A—C3A—H3A	120.00
N1—S1—C1A	107.9 (3)	C2A—C3A—H3A	120.00
N1—S1—C1B	106.9 (3)	C4B—C3B—H3B	120.00
C10—O3—C13	117.3 (4)	C2B—C3B—H3B	120.00
S1—N1—C7	124.2 (3)	C3A—C4A—H4A	120.00
S1—N1—H1	112 (2)	C5A—C4A—H4A	120.00
C7—N1—H1	115 (3)	C5B—C4B—H4B	120.00
S1—C1A—C2A	122.6 (5)	C3B—C4B—H4B	120.00
S1—C1A—C6A	117.4 (5)	C6A—C5A—H5A	120.00
C2A—C1A—C6A	120.0 (6)	C4A—C5A—H5A	120.00
S1—C1B—C2B	113.8 (7)	C4B—C5B—H5B	120.00
C2B—C1B—C6B	120.0 (8)	C6B—C5B—H5B	120.00
S1—C1B—C6B	126.3 (8)	C1A—C6A—H6A	120.00
C1A—C2A—C3A	120.0 (7)	C5A—C6A—H6A	120.00
C1B—C2B—C3B	120.2 (11)	C5B—C6B—H6B	120.00
C2A—C3A—C4A	120.0 (7)	C1B—C6B—H6B	120.00
C2B—C3B—C4B	119.8 (12)	C9—C8—H8	120.00
C3A—C4A—C5A	120.0 (6)	C7—C8—H8	120.00
C3B—C4B—C5B	120.1 (9)	C8—C9—H9	120.00
C4A—C5A—C6A	120.0 (7)	C10—C9—H9	120.00
C4B—C5B—C6B	120.0 (8)	C12—C11—H11	120.00
C1A—C6A—C5A	120.0 (7)	C10—C11—H11	120.00
C1B—C6B—C5B	120.0 (10)	C11—C12—H12	120.00
N1—C7—C12	119.9 (4)	C7—C12—H12	120.00
N1—C7—C8	120.6 (4)	O3—C13—H13C	109.00
C8—C7—C12	119.4 (4)	O3—C13—H13B	109.00
C7—C8—C9	120.5 (4)	H13B—C13—H13C	109.00
C8—C9—C10	120.4 (4)	H13A—C13—H13B	109.00
O3—C10—C11	115.8 (4)	H13A—C13—H13C	109.00
O3—C10—C9	125.1 (4)	O3—C13—H13A	109.00
C9—C10—C11	119.1 (4)		
			
O1—S1—N1—C7	−173.2 (3)	C2A—C1A—C6A—C5A	0.0 (12)
O2—S1—N1—C7	−43.9 (4)	C1A—C2A—C3A—C4A	−0.1 (14)
C1A—S1—N1—C7	71.8 (4)	C2A—C3A—C4A—C5A	0.0 (13)
O1—S1—C1A—C2A	−11.8 (7)	C3A—C4A—C5A—C6A	0.0 (12)
O1—S1—C1A—C6A	168.6 (6)	C4A—C5A—C6A—C1A	0.0 (12)
O2—S1—C1A—C2A	−142.6 (7)	N1—C7—C8—C9	175.8 (4)
O2—S1—C1A—C6A	37.8 (6)	C12—C7—C8—C9	−0.7 (6)
N1—S1—C1A—C2A	102.3 (7)	N1—C7—C12—C11	−174.4 (4)
N1—S1—C1A—C6A	−77.3 (6)	C8—C7—C12—C11	2.2 (7)
C13—O3—C10—C9	−3.3 (7)	C7—C8—C9—C10	−0.1 (7)
C13—O3—C10—C11	178.3 (4)	C8—C9—C10—O3	−178.9 (4)
S1—N1—C7—C8	67.4 (5)	C8—C9—C10—C11	−0.6 (7)
S1—N1—C7—C12	−116.2 (4)	O3—C10—C11—C12	−179.4 (4)
S1—C1A—C2A—C3A	−179.6 (7)	C9—C10—C11—C12	2.1 (7)
C6A—C1A—C2A—C3A	0.0 (13)	C10—C11—C12—C7	−2.9 (7)
S1—C1A—C6A—C5A	179.6 (6)		

Symmetry codes: (i) −*x*+1, *y*+1/2, −*z*+1/2; (ii) *x*−1, *y*, *z*; (iii) *x*, *y*+1, *z*; (iv) −*x*+2, *y*+1/2, −*z*+1/2; (v) *x*+1/2, −*y*+3/2, −*z*; (vi) *x*−1/2, −*y*+3/2, −*z*; (vii) *x*+1, *y*, *z*; (viii) *x*+1/2, −*y*+5/2, −*z*; (ix) −*x*+1, *y*−1/2, −*z*+1/2; (x) *x*−1/2, −*y*+5/2, −*z*; (xi) −*x*+2, *y*−1/2, −*z*+1/2; (xii) *x*, *y*−1, *z*.

### Hydrogen-bond geometry (Å, °)

**Table:**

Cg1, Cg2 and Cg3 are the centroids of the C1A–C6A, C7–C12 and C1B–C6B rings, respectively.

**Table:**

*D*—H···*A*	*D*—H	H···*A*	*D*···*A*	*D*—H···*A*
N1—H1···O2^vii^	0.83 (2)	2.22 (2)	3.039 (4)	170 (4)
C8—H8···Cg2^x^	0.93	2.93	3.613 (5)	132
C13—H13B···Cg1^vi^	0.96	2.98	3.766 (6)	140
C13—H13B···Cg3^vi^	0.96	2.96	3.763 (7)	143

Symmetry codes: (vii) *x*+1, *y*, *z*; (x) *x*−1/2, −*y*+5/2, −*z*; (vi) *x*−1/2, −*y*+3/2, −*z*.
